# Longer work experience and age associated with safety attitudes in operating room nurses: an online cross-sectional study

**DOI:** 10.1136/bmjoq-2022-002182

**Published:** 2024-01-11

**Authors:** Anette Nyberg, Birgitta Olofsson, Ami Fagerdahl, Michael Haney, Volker Otten

**Affiliations:** 1 Department of Nursing, Faculty of Medicine, Umeå University, Umeå, Sweden; 2 Department of Surgical and Perioperative Sciences, Anesthesiology, Faculty of Medicine, Umeå University, Umeå, Sweden; 3 Department of Surgical and Perioperative Sciences, Orthopedics, Faculty of Medicine, Umeå University, Umeå, Sweden; 4 Department of Clinical Research and Education, Södersjukhuset, Karolinska Institutet, Stockholm, Sweden

**Keywords:** Attitudes, Nurses, Patient safety, Safety culture, Surgery

## Abstract

**Background:**

Patient safety is fundamental when providing care in the operating room. Still, adverse events and errors are a challenge for patient safety worldwide. To avoid preventable patient harm, organisations need a positive safety culture, the measurable component of which is known as the safety climate. To best improve the safety climate the current attitudes to safety must first be understood.

**Aim:**

To explore operating room nurses’ safety attitudes and their views on how to improve patient safety in operating rooms.

**Method:**

A cross-sectional study using the Swedish-translated version of the Safety Attitudes Questionnaire, Operating Room version. Data were collected using an online survey platform.

**Results:**

358 operating room nurses completed the questionnaire. The results show that the older age group rated their working conditions and management support as better than the younger age groups. The older age group also rated their stress recognition as lower compared with the younger age groups. The same pattern was seen in terms of work experience, with more-experienced respondents showing a higher mean score for the factor working conditions and a lower mean score for the factor stress recognition as compared with their less-experienced colleagues. When comparing hospital types, county hospital employees had higher factor scores for safety climate, job satisfaction and working conditions than university hospital employees. The respondents’ most recurring recommendations for improving patient safety were ‘Having better and clearer communication’ followed by ‘Having enough time to do things the way they should be done’.

**Conclusion:**

More focus on safety with increasing age and experience was observed in this cohort. Need for improvements is reported for patient safety in operating rooms, mainly when it comes to communication and workload. To improve and develop patient safety in the operating room, the organisational safety climate needs to be actively managed and developed. One step in actively managing the safety climate may be efforts to retain experienced operating room nurses.

**WHAT IS ALREADY KNOWN ON THIS TOPIC**

- To improve the safety climate in the operating room, current safety attitudes need to be understood. There is little data on operating room nurses’ safety attitudes specifically and their views on how to improve patient safety in operating rooms.

**WHAT THIS STUDY ADDS**

- Longer working experience and older age are shown as related to operating room nurses’ safety attitudes. The operating room nurses surveyed here expressed that improvements were needed to ensure patient safety in their workplaces, above all when it comes to communication and workload.

**HOW THIS STUDY MIGHT AFFECT RESEARCH, PRACTICE OR POLICY**

- Efforts, such as perceptions of management, working conditions and teamwork, must be managed actively to retain operating room nurses and minimise risks in the operating room that can contribute to preventable patient complications.

## Background

Patient safety is fundamental when providing medical care. Preventable complications can arise in form of adverse events, errors and inherent risks related to healthcare, and minimising these is still a major challenge.^1^ According to a report from the Swedish National Board of Health and Welfare, 30% of care-related injuries that lead to permanent harm or death occur during perioperative care, and 39% of these have been judged to be preventable.^2^ Twenty years ago, the Institute of Medicine declared that healthcare organisations must develop and enhance their safety culture^3^ since a positive safety culture is fundamental to avoiding preventable patient injury.^4 5^ The terms safety culture and safety climate are sometimes used interchangeably.^6^ Safety culture is defined as a complex concept reflecting essential values, norms and expectations, while safety climate is defined as the staff’s views, attitudes and beliefs about safety and risks.^7^ Thus, safety climate is the more appropriate term to use when measuring attitudes to safety.^8^ Changeability of risk related to task, work environment and staff behaviour can potentially be predicted by measuring frontline workers’ safety attitudes, thereby providing leaders more opportunity to address the organisational cultural barriers.^9 10^

Safety climate, including safety attitudes, is an assessable component of an organisation’s safety culture. An appropriate and positive safety climate is needed for an organisation to learn from errors and adverse events, and thereby improve its processes to achieve and maintain a high level of patient safety. A poor safety climate has been linked to increased rates of adverse events and readmissions,^11^ while a positive patient safety climate has been linked to higher safety performance.^12^ Questionnaires can be used to assess frontline workers’ attitudes and perceptions relevant to the safety of the operating room (OR).^13^ Survey findings concerning patient safety climate in healthcare organisations have been reported, and some results have been published supporting connections between safety climate and patient outcomes.^12 14 15^ Previous studies using the Safety Attitudes Questionnaire, Operating Room version (SAQ-OR), have largely compared safety attitudes among various OR professionals.^14 16 17^ The results from two such studies showed differences in the perception of teamwork in the OR between physicians and nurses^14^ and between physicians and non-physicians,^16^ while another study showed no differences between professions in terms of teamwork.^17^

The safety of healthcare can be investigated and assessed within the systems where errors and adverse events occur most frequently.^13^ The OR is described both as a high-risk environment for patients and as a complex workplace.^18^ Several risk factors influence clinical practice, including the organisation’s safety climate, staffing levels and workload.^19^ Factors that operating room nurses (ORNs) perceive as threats to patient safety in the OR include understaffing^20^ and an increased working pace.^21 22^ Many countries are currently experiencing a shortage of specialist nurses, including in perioperative settings, for example, resulting in more pressure for getting procedures done despite incomplete staffing.^23^

Current attitudes concerning safety in the healthcare workplace need to be understood in order to further improve the safety climate. The present study aimed to explore ORNs’ safety attitudes and their views on how to improve patient safety in ORs.

## Methods

### Study design

The study was an online cross-sectional survey on the safety attitudes among ORNs employed at public hospitals in Sweden.

### Safety Attitudes Questionnaire, operating room

The SAQ-OR is a validated instrument used in the USA and Europe,^7 24^ to measure attitudes across six factors: safety climate, teamwork climate, job satisfaction, working conditions, stress recognition and perceptions of management.^13^ The Swedish SAQ-OR version consists of 57 items, with 30 addressing the six factors of safety climate (supplemental material SAQ-OR Swedish version).^24^

Each item is scored on a 5-point Likert scale from 1 (strongly disagree) to 5 (strongly agree), with 6 being not applicable. In cases with missing values, the factor mean score was calculated from items with valid values. The mean scores of the factors were calculated after converting scores to a 100-point scale (1=0, 2=25, 3=50, 4=75, 5=100 and 6=0).^13 17^ As in the original SAQ, ‘not applicable’ was included as a response alternative and calculated as no score, the same for a missing value.^13 17^ Two items with reverse anchoring were re-coded before analysis. The percentage of respondents who had positive attitudes toward safety (75 or higher on the 100-point scale) was used to calculate the positive response rate.^24^ The SAQ-OR also include an open question for recommendations to improve patient safety at the respondent’s workplace.^13^

### Setting

The OR team in Sweden generally consists of a surgeon, an anaesthesiologist, a nurse anaesthetist, an ORN and an assistant nurse. All team members work interdependently performing complex and diverse tasks,^25^ and all are crucial to the team’s performance and the reduction of potential error and patient harm. Swedish ORNs are responsible for patient positioning, skin disinfection and sterile draping, and ensuring that instruments and implants are in place.^26^ To work as an ORN in Sweden, a registered nurse (RN) must attend a 1-year OR nursing specialist programme that is concluded with an academic Master’s degree.^27^

### Participants

The inclusion criteria for this study were all public hospital employed ORNs. In 2019, the number of ORNs employed by Swedish public hospitals was 2323, including both university and county hospitals.^28^ For this study, all heads of OR departments in public hospitals in Sweden were contacted with a request to provide the email addresses of their currently employed ORNs. Exclusion criteria were ORNs at that time employed by private hospitals and staffing agencies. Fifteen out of 21 county councils in Sweden provided in total 1441 email addresses of their ORNs and 358 ORNs completed the questionnaire. Forty-four recipients completed only part of the survey, and these were not included in the final analysis.

### Data collection

The data collection took place during an ongoing global pandemic. Healthcare in Sweden, as healthcare globally, needed to relocate its staff resources to enable an expansion of hospital beds in intensive care. By cancelling elective surgery, the staff of OR departments were temporarily relocated to work in intensive care wards or postoperative wards.

For data collection, the Swedish-translated version of SAQ-OR was used.^24^ Data were collected from July to November 2021 using an online survey platform (Artologik: Survey & Report, Artisan Global Media, Sweden). An email with information about the study purpose and an invitation to complete the questionnaire was sent to the provided addresses of ORNs. All responses were handled confidentially; only one person in the research group (AN) knew how to connect a particular response to the correct email address. Non-responders received up to three reminders. The questionnaire was estimated to take about 20 min to complete.

In addition to SAQ-OR responses, this study also evaluated demographic variables such as sex, age group, type of hospital that the ORNs currently worked at, work experience as an ORN and surgical speciality. To ensure data quality and integrity the data set was cleaned, coded, and verified before they were entered into SPSS.

### Data analysis

A calculation for sample size showed that for reliable analysis of 30 items addressing the six safety attitude factors and with 10-fold scale items, at least 300 completed surveys would be needed. Since the data were distributed normally, they are presented as ordinal rank means and a 95% CI. One-way analysis of variance was used to test for differences in factor means separately across age groups and experience groups. Bonferroni correction was used for multiple comparisons, to explore between which age and experience groups there were significant differences in group means. An independent group t-test was used to compare university and county hospital factor means. A p value<0.05 was considered statistically significant. To evaluate the correlation between age and experience, Kendall’s tau-b analysis was employed, and the multicollinearity between these variables was also examined. The SAQ factor score was treated as an ordinal scale variable. All statistical analyses were performed using SPSS V.27 (IBM, USA).

Open answers were evaluated using thematic analysis,^29^ an adaptable method to interpret qualitative data and review key features in a data set. Building subthemes in the presence of recurring recommendations enabled a deeper understanding of respondents’ views on what is needed to improve patient safety in their workplace. The six steps of thematic analysis included the following familiarisation with data; initial coding; searching for themes; reconsidering themes; defining and naming themes; and creating the report.^29^ An inductive coding reliability approach was used to perform the thematic analysis using MAXQDA 2020 (VERBI Software, Germany). Thematic analysis here involved pattern recognition to identify recurring patterns and generate a number to represent each qualitative outcome, as numbers are an integral part of qualitative research.^30^

## Results

The questionnaire was completed by 358 ORNs, resulting in an overall response rate of 25%. Of the 358 ORNs responding, 80% specified that they work 80% or more clinically as frontline workers. The respondents were geographically dispersed throughout Sweden. Further detailed demographic variables are shown in table 1.

**Table 1:** Demographics of respondents to SAQ-OR

Sex	
Female	340 (95)
Male	18 (5)
Type of hospital	
University hospitals	173 (48)
Other hospitals	151 (42)
Type of hospital unknown	34 (9)
Working within one specialty or several specialties	
One specialty	155 (43)
Several specialties	200 (56)
Unknown	3 (1)
Age groups	
20–39 years	74 (21)
40–49 years	93 (26)
50–59 years	133 (37)
60–69 years	58 (16)
Years of specialty experience	
Mean	18
95% CI	17–19
Years of specialty experience	
Less than 10	113 (32)
10 to 19	92 (26)
More than 20	153 (43)

Number of respondents (%).

SAQ-OR, Safety Attitudes Questionnaire, Operating Room version.

Table 2 contains the mean factor scores and missing data for each factor in SAQ-OR. The factor job satisfaction had the highest mean score, and perception of management had the lowest.

**Table 2:** Mean scores for each factor in SAQ-OR

Factor	Number of items	N (missing)	Mean	95% CI
Safety climate	7	352 (6)	72	70 to 73
Teamwork climate	6	347 (11)	74	73 to 76
Job satisfaction	5	354 (4)	81	79 to 82
Working conditions	4	316 (42)	67	65 to 69
Stress recognition	4	349 (9)	62	59 to 64
Perceptions of management	4	323 (35)	58	56 to 60

Parentheses denote the number of missing or incomplete responses.

N, number of complete responses; SAQ-OR, Safety Attitudes Questionnaire, Operating Room version.

The proportion of respondents who reported positive scores for each factor is presented in table 3. The percentage of positive scores for the factors ranged from 26% to 73%. Within factors with limited positive responses, such as perception of management, the item ‘The levels of staffing in this clinical area are sufficient to handle the number of patients’ had 28% positive responses. Within the factor working conditions, items ‘Problem personnel is dealt with constructively by our hospital management’ and ‘All necessary information for diagnostic and therapeutic decisions is routinely available to me’ had 37% and 40% positive responses, respectively.

**Table 3:** Distribution of positive scores for each factor of SAQ-OR

Factor	Positive
Safety climate	49% (174)
Teamwork climate	60% (216)
Job satisfaction	73% (261)
Working conditions	44% (156)
Stress recognition	37% (131)
Perception of management	26% (93)

Percentage (proportion) of respondents reporting a positive score for each factor of SAQ-OR. Respondents were regarded as having a positive score if the score was 75 or greater.

SAQ-OR, Safety Attitudes Questionnaire, Operating Room version.

Differences in SAQ-OR factor mean scores, by age, experience and hospital type are presented in table 4.

**Table 4:** Differences in SAQ-OR factor mean scores by age, experience and hospital type

	N	Safety climate	Teamwork climate	Job satisfaction	Working conditions	Stress recognition	Perception of management
Age groups							
20–39 years	74	72 (68 to 75)	74 (71 to 78)	79 (75 to 82)	66 (62 to 70)	69 (65 to 73)	57 (53 to 61)
40–49 years	93	70 (67 to 74)	73 (69 to 76)	81 (78 to 84)	63 (59 to 66)	64 (59 to 68)	55 (51 to 59)
50–59 years	133	72 (69 to 74)	75 (72 to 77)	81 (79 to 83)	67 (64 to 71)	61 (57 to 65)	59 (55 to 62)
60–69 years	58	74 (70 to 77)	76 (73 to 80)	83 (80 to 86)	73 (70 to 76)	50 (45 to 56)	64 (60 to 69)
P value*		0.572	0.539	0.387	0.006	<0.001	0.019
Years of experience							
Less than 10	113	71 (67 to 74)	74 (71 to 77)	80 (77 to 82)	65 (61 to 68)	66 (63 to 70)	57 (53 to 60)
10 to 19	92	70 (67 to 73)	72 (69 to 76)	80 (77 to 82)	65 (62 to 68)	66 (61 to 70)	57 (53 to 60)
20 or more	153	73 (71 to 76)	76 (74 to 78)	82 (80 to 84)	70 (67 to 72)	56 (52 to 59)	60 (57 to 63)
P value*		0.145	0.206	0.183	0.025	<0.001	0.148
Type of hospital							
University hospital	173	69 (67 to 71)	73 (70 to 75)	79 (77 to 81)	64 (61 to 67)	60 (57 to 63)	57 (54 to 60)
County hospital	151	73 (71 to 76)	75 (73 to 78)	82 (80 to 84)	70 (67 to 72)	62 (59 to 65)	59 (56 to 62)
P value†		0.016	0.107	0.035	0.003	0.437	0.325

Safety Attitudes Questionnaire, Operating Room version (SAQ-OR) factors presented as mean (95% CI) comparing four age groups, three groups of experience and two types of hospitals.

*The one-way analysis of variance (ANOVA) comparison was made between the different age subgroups for each SAQ-OR factor to test if the groups differed from each other. One-way ANOVA comparison was also made between subgroups for years of experience.

†An independent t-test was made for comparison between the two hospital types for each SAQ-OR factor.

Post hoc comparisons using Bonferroni are reported with a mean difference (MD), 95% CI and p value. Post hoc comparison indicated that for factor working conditions, the mean score for ORNs aged 60 years and older was higher compared with ORNs aged 40–49 years (MD 9.90, 95% CI (2.41 to 17.40), p=0.003). ORNs aged 60 years and older had a higher mean score for perception of management compared with ORNs aged 40–49 years (MD 9.32, 95% CI (1.29 to 17.36), p=0.019). The oldest age group of ORNs had a lower mean for stress recognition than all the younger age groups. The oldest age group compared with the 20–39 years age group (MD −18.49, 95% CI (−28.13 to −8.85), p<0.001). The oldest age group compared with the 40–49 years age group (MD −13.28, 95% CI (−22.48 to −4.08), p<0.001). The oldest age group compared with the 50–59 years age group (MD −10.64, 95% CI (−19.29 to −1.99), p=0.007).

ORNs with work experience of 20 years or more had a higher factor mean score for working conditions compared with ORNs with less than 10 years of experience (MD 5.12, 95% CI (0.05 to 10.18), p=0.047). ORNs with work experience of 20 years or more had also a lower factor mean score for stress recognition compared with both the younger experience groups. Specifically, ORNs with 20 years or more of experience group compared with less than 10 years of experience group (MD −10.45, 95% CI (−16.68 to −4.22), p<0.001) The most experienced group compared with the 10–19 years of experience group (MD −9.69, 95% CI (−16.31 to −3.06), p=0.001). A test of correlation revealed a statistically significant positive correlation between age and years of experience, with Kendall’s tau-b 0.599 (p<0.001). A test of multicollinearity between the same variables showed a variance inflation factor of 1.811.

In comparison between hospital types, county hospital-employed ORNs had higher factor means for safety climate, job satisfaction and working conditions than university hospital-employed ORNs.

249 of the 358 respondents (69%) answered the open question: ‘What are your three most important recommendations for improving patient safety at your workplace?’ The open answers varied in scope, from single words to long descriptions. Five main themes and 15 subthemes emerged from the thematic analysis (table 5), suggesting several areas to improve patient safety at the respondents’ workplaces. The most common recommendation for improving patient safety was ‘Having better and clearer communication’ from the theme *Developing teamwork and performance*, followed by ‘Having enough time to do things the way they should be done’ from the theme *Improved working conditions*.

**Table 5:** Themes, subthemes and frequency of mention in the open answers in SAQ-OR

Themes	Subthemes	Frequency of mention
Developing teamwork and team performance	Having better and clearer communication	93
	Having better collaboration and opportunity for continuous training	77
	Having openness and good team spirit	35
Improved working conditions	Having enough time to do things the way they should be done	85
	Having adequate staffing level	49
	Having competent and experienced staff	43
	Having a permissive working environment	33
	Having better working hours	14
Taking professional responsibility	By following guidelines	79
	Having responsible staff	13
Effective use of information	A better flow of information and opportunity for feedback	76
	Improvement of surgical planning	27
Leadership that meets expectations and supports patient safety	Leadership being competent and committed	15
	Leadership being supportive	15
	A clear leadership focusing on safety	13

N=249, number of respondents who answered the open question ‘What are your three most important recommendations for improving patient safety at your workplace?’

SAQ-OR, Safety Attitudes Questionnaire, Operating Room version.

## Discussion

This study observed an improved focus on safety with both increasing age and experience. The responses showed that ORNs perceived that improvements are needed to ensure patient safety in their workplaces, above all when it comes to communication and workload. The county hospital employees rated their safety climate, job satisfaction and working conditions higher than did university hospital employees.

As expected, a correlation was observed between age and experience group variables though a multicollinearity assessment supports that the two variables can be interpreted independently. An independent influence of one factor was apparent in certain SAQ-OR factors. When looking at age groups, ORNs aged 60 years and older reported a higher factor mean score for working conditions compared with their younger colleagues, supporting the idea that older ORNs can be more satisfied with their working conditions than younger ORNs. This result agrees with a previous study that showed that nurses assessed their level of competence as higher the older and more experienced they were,^31^ and felt more confident at work. However, this could also be explained by the idea that older nurses professionally distance themselves as they experience being controlled by their work situation and lack the authority to act.^32^

ORNs with work experience of 20 years or more reported a higher factor mean score for working conditions than the less experienced ORNs. This indicates that ORNs with more experience had better preconditions for a good safety attitude compared with those with less, suggesting that having more experienced nurses in OR departments may positively influence the safety climate. This is in line with a previous study showing that retaining experienced nurses is crucial in maintaining specialist expertise in emergency and critical care settings to ensure safe care for patients.^33^

There is always a potential risk for complacency or hierarchy effects with more experienced staff. Still, integrating new or less experienced ORNs into the OR is crucial. A previous study on newly trained ORNs emphasised the importance of providing support and guidance during their transition to becoming ORNs.^34^ A well-functioning team was reported as a vital factor in their successful transition, fostering a sense of visibility, security and comfort among newly trained ORNs. The absence of such support could explain why less experienced ORNs in the current study rated working conditions factor significantly lower and stress recognition factor higher compared with their more experienced colleagues.

More predictable working conditions could decrease workload and stress in the OR. The present results show that older and more experienced ORNs are less affected by stress than younger or less experienced ORNs. According to a previous study carried out in intensive care, a demanding work environment with little ability to control the situation and receive support can mean more stress for the nurses, which in turn can affect patient safety.^35^ It has also been suggested that the occasional use of more dedicated teams could be a way to reduce stress.^36^ Furthermore, from a human factors perspective, it has been shown that improving work-life balance for individual workers can also help to improve teamwork and safety climate, and thus patient care.^37^ The optimal work-life balance can depend on an individual worker’s professional role and the length of time in the speciality.^38^

There were differences in attitudes to safety between employees of university hospitals and those of county hospitals. County hospital-employed respondents had higher factor mean scores for safety climate, job satisfaction and working conditions compared with university hospital-employed respondents. Since high-risk operations are often carried out at university hospitals, it would be important to improve these factors there. This can also reflect current conditions in Sweden where particularly university hospitals steadily contend with nursing staffing problems.

One possible explanation for the higher safety climate score in county hospitals could be that they have smaller OR units and may therefore have more team familiarity compared with the larger OR units in university hospitals. In an earlier study, consistency as in familiarity with team members and stability in teams was found to promote safety by combining team members’ different skills in long-term planning.^39^ Team cohesiveness is seen as a valued and important factor for team performance^40^ and has been linked with shorter surgical time, fewer surgical errors and disruptions, less miscommunication and fewer patient readmissions.^36 41^ In a previous study implementing a comprehensive patient safety programme, an association was found between increased scores of teamwork and safety climate and a decrease in patient harm and mortality.^42^ It is possible that county hospital-employed respondents scored their job satisfaction higher because they have worked longer at their current workplace than university hospital-employed respondents. Indeed, higher levels of job satisfaction have been reported by nurses who have worked longer in a specific unit or hospital in acute settings.^43^

In the open answers results, there was expression of a perceived need for improved working conditions in the OR. Respondents underscored the importance of adequate staffing levels with competent, experienced and permanent staff, as well as a permissive working environment, improving safety. To prevent errors in the OR a good working environment is required, including good planning and management that assembles enough experienced OR teams for the various procedures.^39^ Frustration can occur for frontline workers striving to eliminate harm when there is a gap between expected standards and provided care.^44^ Therefore, institutions can strategically focus on equipping frontline workers with risk management strategies when joint standards cannot be reached and patient safety is compromised. The ORNs expressed that management did not seem supportive of a focus on patient safety. To enable lasting improvements in both patient and workforce safety, leadership should be committed and recognise the importance of safety.^45^

Many ORNs reported ‘Having enough time to do things as they should be done’ as a suggestion for improving safety in their workplace. The expressed perception of time constraints indicates that workers may frequently operate near the limit of safety margins. In addition, new situations can arise on top of that which acutely increases stress and further compromises patient safety. A study by Wheelock *et al* exploring the relationship between distractions, stress, workload and teamwork in the OR revealed that distractions occurred regularly,^46^ and reported that while some distractions in the OR may be unavoidable, many could be limited through a systematic approach and thereby also limit the risk of error.

The subtheme ‘Having better and clearer communication’ within the main theme ‘Developing teamwork and team performance’ was most frequently mentioned in responses about how patient safety can be improved. There is potential to improve team communication, and therefore safety, by using a surgical checklist.^47^ In an earlier intervention study, the introduction of the WHO surgical checklist increased teamwork and safety climate scores post intervention, which correlated with a reduction in postoperative morbidity and mortality.^15^

In the Swedish context, management of specialty OR functions is a formal administrative position. In this study, the vast majority of the 358 responding ORNs specified that they work 80% or more clinically in OR. In a previous study, senior managers perceived patient safety climate more positively than managers on a lower level.^48^ Singer *et al* also indicated that senior managers not frequently exposed to frontline work need support to gain knowledge of work systems to improve safety successfully.

Safety climate survey results can aid patient safety dialogues within an organisation and indicate where more work is needed.^7^ In this study, positive scores varied from 26% to 73% across the six factors. For the safety climate to be considered ‘good’, one expert group suggests that 80% of respondents should report positive scores.^49^ Good leadership implies that management shares the staff’s responsibility for patient safety and provides support for safe working conditions.^17^ Only 26% of respondents reported a positive score for the factor perception of management. These findings are in line with the perception that to develop and foster a culture of safety, leaders must support and promote it daily.^50^ Leape *et al* visualise a culture that is grounded in task and purpose, with teamwork at its core and organisational managers holding themselves responsible for safety and learning to improve.^51^

### Strengths and limitations

A strength of this study is that there was a variation among the respondents in terms of geographical distribution, including between both major city areas and areas with medium-sized cities. The distribution between men and women among the respondents was representative of the profession in Sweden. Of the 2323 ORNs employed by Swedish public hospitals in 2019, 94% were women.^28^ The open question included in the questionnaire and its high response rate, provide valuable information about how to improve safety in ORs, and these supporting results were a strength.

Some limitations in the study design include the low overall response rate, which means that we cannot generalise with confidence to the whole population, though this exploratory analysis can be informative. Still, 358 ORNs responded despite the data collection taking place during a global pandemic. Data collection occurring during the COVID-19 pandemic with a very overburdened healthcare system potentially limited inclusion to the study.

### Conclusion

Longer working experience and older age were associated with ORNs’ attitudes to safety. According to the ORNs in this study, further improvements are needed to ensure patient safety in their workplaces, mainly with regard to communication and workload. To improve and develop patient safety in the OR, the organisational safety climate needs to be actively managed and developed. One step in actively managing the safety climate can be efforts to retain experienced operating room nurses.

## Data Availability

Responses, analysis and unpublished data from this study are securely stored and only available to AN and can be shared if a reasonable request is submitted to the authors and Umeå University.
